# Genome-wide profiling of DNA methylation and gene expression in *Crassostrea gigas* male gametes

**DOI:** 10.3389/fphys.2014.00224

**Published:** 2014-06-17

**Authors:** Claire E. Olson, Steven B. Roberts

**Affiliations:** School of Aquatic and Fishery Sciences, University of WashingtonSeattle, WA, USA

**Keywords:** epigenetics, DNA methylation, oyster, bivalves, gene expression

## Abstract

DNA methylation patterns and functions are variable across invertebrate taxa. In order to provide a better understanding of DNA methylation in the Pacific oyster (*Crassostrea gigas*), we characterized the genome-wide DNA methylation profile in male gamete cells using whole-genome bisulfite sequencing. RNA-Seq analysis was performed to examine the relationship between DNA methylation and transcript expression. Methylation status of over 7.6 million CpG dinucleotides was described with a majority of methylated regions occurring among intragenic regions. Overall, 15% of the CpG dinucleotides were determined to be methylated and the mitochondrial genome lacked DNA methylation. Integrative analysis of DNA methylation and RNA-Seq data revealed a positive association between methylation status, both in gene bodies and putative promoter regions, and expression. This study provides a comprehensive characterization of the distribution of DNA methylation in the oyster male gamete tissue and suggests that DNA methylation is involved in gene regulatory activity.

## Introduction

DNA methylation is an important epigenetic process that varies in genomic distribution and biological function across taxa. DNA methylation involves the addition of a methyl group to a cytosine pyrimidine ring and most often occurs as part of C-G nucleotide pairs, frequently referred to as CpG dinucleotides. Mammals exhibit a pattern commonly referred to as global methylation, in which 70–80% of CpG dinucleotides are methylated (Bird, 1980). In contrast, invertebrates display relatively low levels of DNA methylation, from almost no methylation in *Drosophila melanogaster* (Gowher et al., 2000) to intermediate levels in the sea urchin *Echinus esculentus* (Bird et al., 1979). In mammals, a primary function of DNA methylation is to suppress gene expression through increased promoter DNA methylation (Bell and Felsenfeld, 2000). However, the function of DNA methylation in invertebrates is variable and likely differs among invertebrate taxa. The roles of methylation include regulation of transcriptional activity (Suzuki and Bird, 2008), alternatively exon splicing (Lyko et al., 2010), and developmental activity (Riviere et al., 2013).

While in general there is a limited amount of comprehensive information regarding DNA methylation in non-mammalian taxa, some recent studies have focused on DNA methylation in the Pacific oyster. The Pacific oyster is an excellent model for studying epigenetic modifications because its life history characteristics make it an important aquaculture species (Glude and Chew, 1982) and genomic resources for this species have recently become available (Zhang et al., 2012). Gavery and Roberts (2010) first reported the presence of DNA methylation in the Pacific oyster. In the same study, *in silico* analysis revealed a significant correlation between gene function and methylation level (Gavery and Roberts, 2010). The relationship between gene methylation and gene function was experimentally corroborated with high-throughput sequencing and it has been proposed that limited methylation in select genes may contribute to increased phenotypic plasticity in highly fluctuating environments (Roberts and Gavery, 2012). More recently, methylation enrichment and bisulfite sequencing were used to describe high-resolution DNA methylation patterns in pooled oyster gill tissue (Gavery and Roberts, 2013). A characterization of DNA methylation during oyster larval development has also been performed, revealing that DNA methylation varies through early development and treatment with 5-Aza-cytidine, a DNA methyltransferase (DNMT) inhibitor, leads to developmental alterations (Riviere et al., 2013). In the same study, researchers found an inverse correlation between methylation proximal to the transcription start site and expression of hox genes (Riviere et al., 2013). In addition to studies that investigate putative function of DNA methylation, other research has begun to evaluate relationships between epigenetic and genetic variations in *C. gigas* mass selection procedures (Jiang et al., 2013).

While a better understanding of DNA methylation is emerging for this species, there are still several questions that remain. Importantly we still do not fully understand the relationship between DNA methylation and gene expression, nor DNA methylation patterns in a single cell type. Examining a single cell type is important as methylation levels and patterns may differ between multiple cell types and life history stages, and our research attempted to limit this potential variability. Spermatozoa are an ideal resource for studying a single cell type and also provide the secondary benefit of understanding more about oyster spermatogenesis. The oyster male gonad consists of numerous gonadal tubules that grow during tissue development (Franco et al., 2008) and evolve according to four successive reproductive stages annually (Berthelin et al., 2000). These gonadal stages include undifferentiated (stage 0), mitosis of spermatogonia and differentiation of germ cells (stage 1), visible spermatogenesis (stage 2), and mature gametes (stage 3) (Franco et al., 2008). This is the first time DNA methylation has been characterized in Pacific oyster gametes, however spermatozoa methylation has been previously examined in other marine invertebrates. For example, spermatozoa DNA methylation has been described in both the marine annelid worm *Chaetopterus variopedatus* (del Gaudio et al., 1997) and *Ciona intestinalis* (Suzuki et al., 2013).

This research represents the first high-resolution characterization of DNA methylation patterns from a single cell type in a mollusc, including an examination of the relationship between gene expression and promoter region methylation. Our results demonstrate that DNA methylation is predominant in intragenic regions (exons and introns) and that there is a positive relationship between methylation and gene expression in *C. gigas*. Furthermore, we were surprised to find similar patterns of tissue-specific methylation in male gametes as has been previously described in oyster gill tissue, thus suggesting that overall methylation levels do not dramatically vary between tissue types, and specifically between gametic and somatic cells.

## Methods

### Bisulfite treated DNA sequencing (BS-Seq)

A single male adult oyster was collected from Thorndyke Bay, WA and thermally conditioned and fed for 6 weeks in the laboratory. Male gamete tissue was scored with a razor blade, gametes rinsed with sterile seawater, centrifuged, and immediately placed on dry ice then stored at −80°C until further processing. Genomic DNA was extracted using DNAzol according to the manufacturer's protocol (Molecular Research Center, Inc. Cincinnati, OH). High molecular weight genomic DNA (6 ug) was used to prepare a library for whole-genome bisulfite sequencing. Lambda DNA (Promega Co. Madison, WI) was added to the sample prior to fragmentation and library construction to serve as a measure of bisulfite conversion efficiency. Extracted DNA was fragmented to an average length of 250 bp using a Covaris S2 (Covaris Inc. Woburn, MA) and fragment size was confirmed by gel electrophoresis. The library was constructed using the Paired-End DNA Sample Prep Kit (Illumina, San Diego, CA) with standard protocols. DNA was treated with sodium bisulfite using the EpiTect Bisulfite Kit (Qiagen, Valencia, CA) and 72 bp paired-end sequencing was performed on the Illumina HiSeq 2000 system. Library construction and sequencing was performed by the High Throughput Genomics Center (htSEQ, Seattle, WA).

DNA sequence reads were mapped to all genomic scaffolds from the *Crassostrea gigas* draft genome (Fang et al., 2012). Sequences were mapped using Bisulfite Sequencing Mapping Program BSMAP v2.73 (Xi and Li, 2009). Resulting data from mapping bisulfite treated reads was analyzed with *methratio*, a Python script that accompanies BSMAP to calculate and extract methylation ratios. Parameters for *methratio* included reporting loci with zero methylation ratios (-z), combining CpG methylation ratios on both strands (-g) and only using unique mappings (-u). The same mapping procedure was also performed with the *Crassostrea gigas* mitochondrial genome (Accession # AF177226). The resulting *methratio* outputs were uploaded to SQLShare (Howe et al., 2011) and queried to examine distribution of methylation. Methylation characteristics were initially calculated for all cytosines.

### Genomic features and CpG dinucleotide methylation

Methylation of CpG dinucleotides was characterized in relation to genomic features. A CpG locus was considered methylated if it had at least 5× coverage and at least half the reads remained unconverted after bisulfite treatment. Methylation ratios were calculated for individual loci as well as for full-length genes and intragenic regions (introns and exons). Methylation on a per gene basis was determined by obtaining the number of methylated cytosines divided by the total number of CpG dinucleotides per region. Genome feature tracks were generated in order to characterize the distribution of methylation in the male gamete tissue. All CpG dinucleotides were identified using the EMBOSS tool fuzznuc (Rice et al., 2000). Methylated CpGs (5× coverage, ≥50% unconverted), sparsely methylated CpGs (5× coverage, 0–50% unconverted) and unmethylated CpG loci (5x coverage, 0% unconverted) within genomic regions were determined using Bedtools (i.e., *intersectBED*) (Quinlan and Hall, 2010). Methylation was examined within exons and introns (Fang et al., 2012), promoter regions (characterized as 1 kb regions upstream from transcription start sites), and putative transposable elements identified using RepeatMasker and the Transposable Element Protein Database (Smit et al., 1996–2010).

### Transcriptome sequencing

Total RNA was isolated using TRI reagent (Molecular Research Center) from the same oyster gamete tissue used for bisulfite sequencing. RNA was enriched for mRNA using Sera-Mag oligo dT beads (Thermo Scientific). A shotgun library was constructed from double stranded cDNA for paired end sequencing by end-polishing, A-tailing and ligation of sequencing adaptors. Sequencing and library preparation were performed on the Illumina HiSeq 2000 platform at the Northwest Genomics Center at the University of Washington (Seattle, WA). RNA-Seq analysis was performed using CLC Genomics Workbench version 6.5 (CLC Bio, Aarhus, Denmark) with high-throughput reads (50 bp paired end) mapped back to the oyster transcriptome (Fang et al., 2012). Initially, sequences were trimmed based on quality scores of 0.05 (Phred; Ewing and Green, 1998; Ewing et al., 1998), and the number of ambiguous nucleotides (>2 on ends). Sequences smaller than 20 bp were also removed. For RNA-Seq analysis, expression values for each gene (28,027) were measured as RPKM (reads per kilobase of exon model per million mapped reads) (Mortazavi et al., 2008) with an unspecific match limit of 10 and maximum number of 2 mismatches.

A Chi-squared test was performed to determine if the degree of gene methylation with respect to gene expression levels (RPKM) was different from what would be expected from a random distribution of methylation levels in promoter regions (*p*-value < 0.05 was considered significant). For promoter region analysis, these regions were determined to be the 1 kb regions upstream from transcription start sites that did not overlap with neighboring genes. In addition, only promoter regions with at least 10 CpG dinucleotides were considered. Oyster genes were classified as either heavily methylated (methylation ratio ≥0.5), sparsely methylated (methylation ratio 0–0.5) or unmethylated (methylation ratio = 0).

## Results

### Bisulfite treated DNA sequencing (BS-Seq)

Bisulfite treated DNA sequence reads (171.5 million) were produced and are available (NCBI Sequence Read Archive: accession number SRX386228). A total of 90 million paired end reads (53% of total reads) and 32 million single end reads (9.6%) mapped to the *Crassostrea gigas* genome. Sodium bisulfite conversion efficiency was estimated to be 99.72% based on analysis of lambda phage DNA. All cytosine dinucleotide motifs were examined and a majority of methylated cytosines were reported in CpG dinucleotides. We found that 15% of the CpG dinucleotides were methylated while the next highest motif (CpA) methylated at 0.14%, which falls within the sodium bisulfite conversion efficiency margin of error (0.28%).

### DNA methylation and genomic features

**Figure 1:**
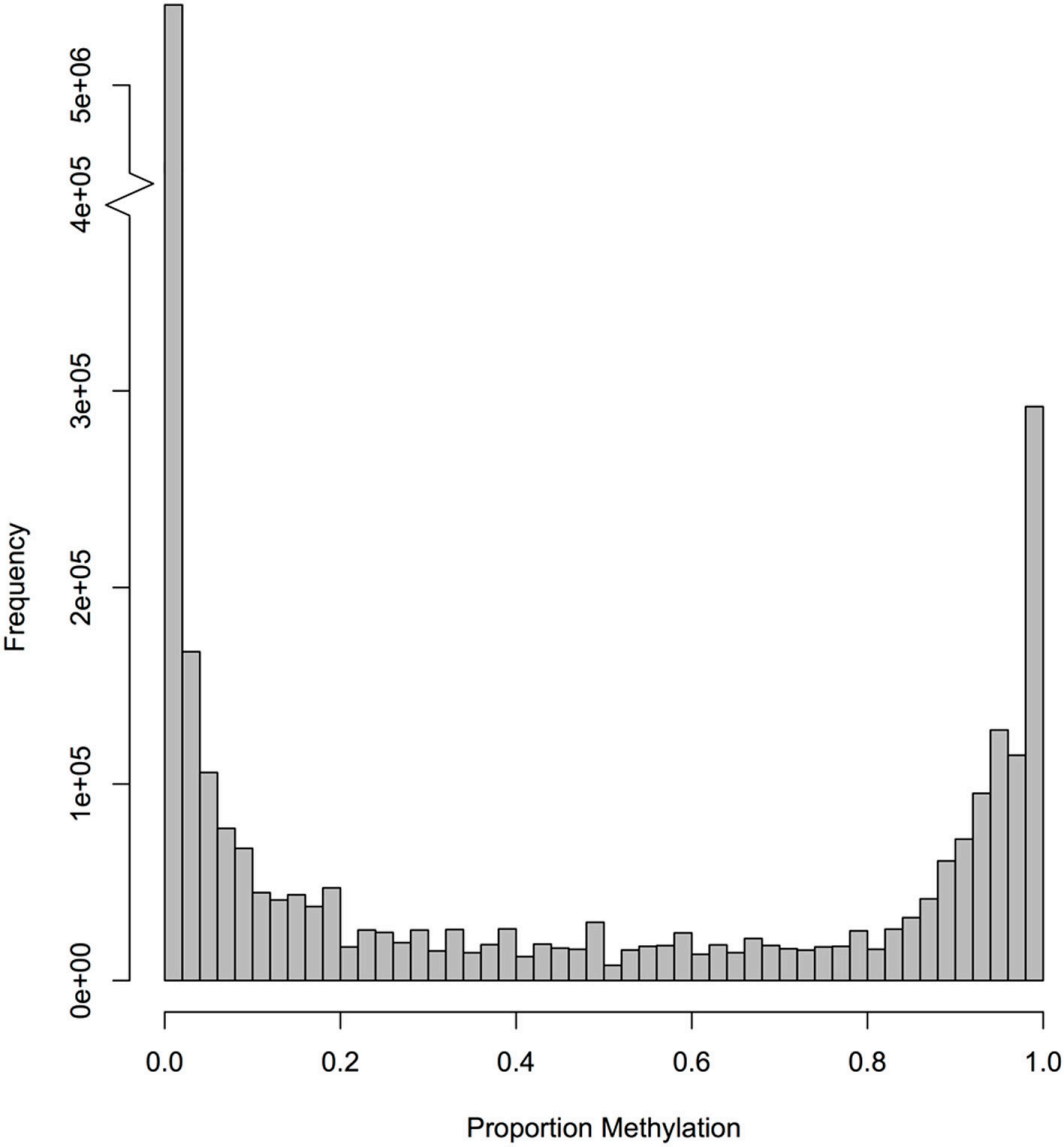
**Frequency of CpG methylation ratios in *C. gigas* male gamete tissue**. A total of 7,642,816 CpG dinucleotides with at least 5× coverage were examined.

The bisulfite sequencing effort provided ≥1x coverage for 8.52 million of the 9.98 million CpGs (85%) in the oyster nuclear genome. Using a 5x coverage threshold, which corresponds to 7.64 million CpGs (77%), the majority of CpGs were not methylated (Figure 1).

**Figure 2:**
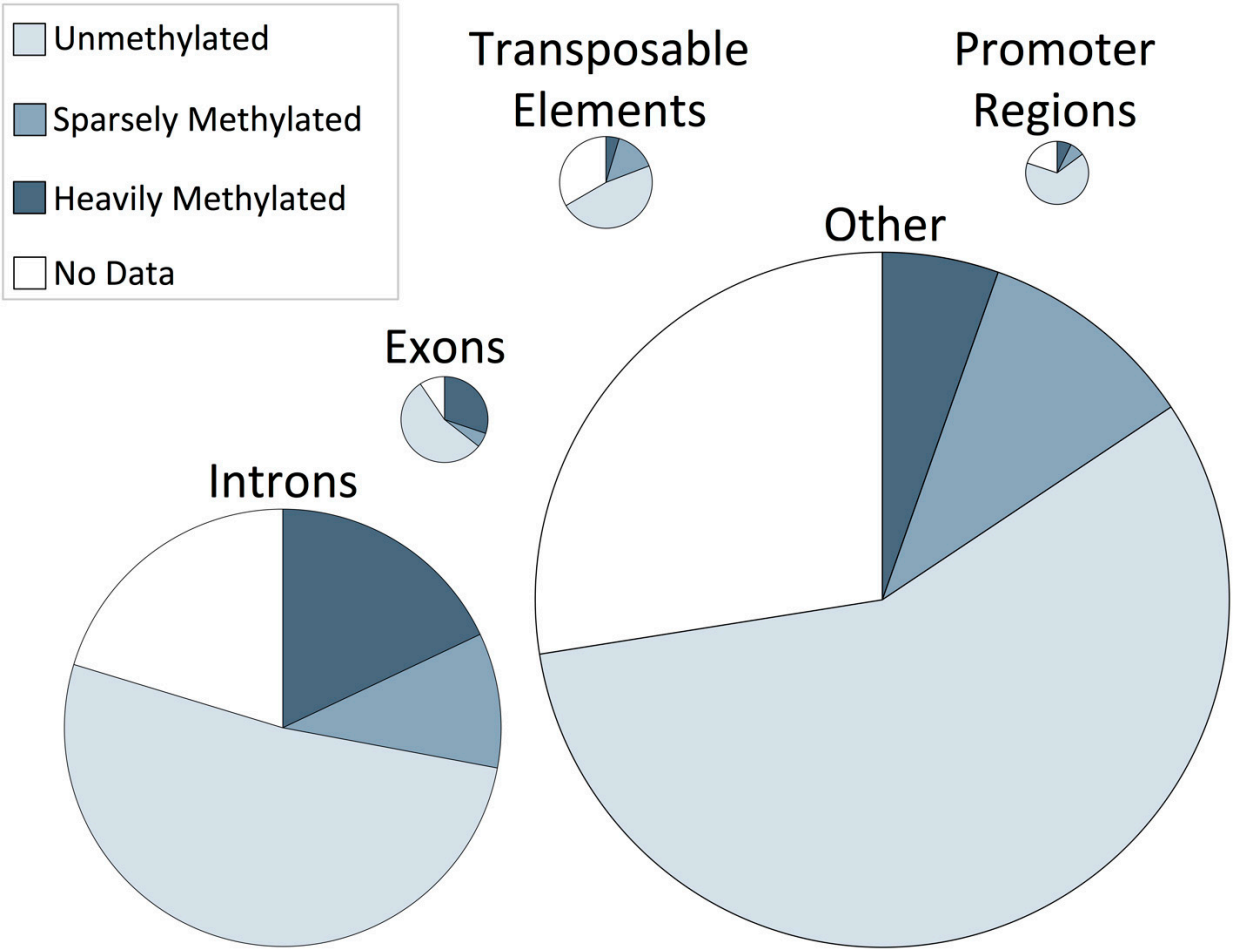
**Genome-wide distribution of CpG methylation in *C. gigas* male gamete tissue within genomic regions**. The proportion of CpG unmethylated (ratio = 0), sparsely methylated (ratio between 0 and 0.5), heavily methylated (ratio ≥0.5), or CpG dinucleotides with less than 5× coverage (“No data”) are shown as percentage contributions to specific oyster genomic regions. Genomic regions are scaled according to their relative CpG contribution to the genome.

The proportion of CpG methylation occurring in specific regions of the oyster genomic landscape were characterized. Methylation occurs predominantly in intragenic regions, with 74% of methylated CpGs found in exons and introns. A total of 30% of CpGs in exons were methylated and 18% of CpGs in introns were methylated (Figure 2). These are particularly high levels of methylation when compared to methylation levels of other oyster genomic regions, wherein between 4 and 7% of CpGs were methylated.

The oyster mitochondrial genome is predominantly unmethylated. With an average coverage of 39.76-fold, of the 2518 cytosines with at least 5x coverage, 2316 cytosines were converted upon bisulfite treatment and no cytosines were considered methylated.

### Whole transcriptome sequencing expression patterns

After quality trimming, 50.3 million reads (paired end 50 bp) remained (NCBI Sequence Read Archive: accession number SRX390346). Expression (RPKM) was detected in a majority of the genes (17,093 genes or 63%). Median expression level was 0.749 and expression level ranged from 0 to 35637.3 RPKM. The relationship between gene methylation and expression levels was examined by determining methylation on a per gene basis.

**Figure 3:**
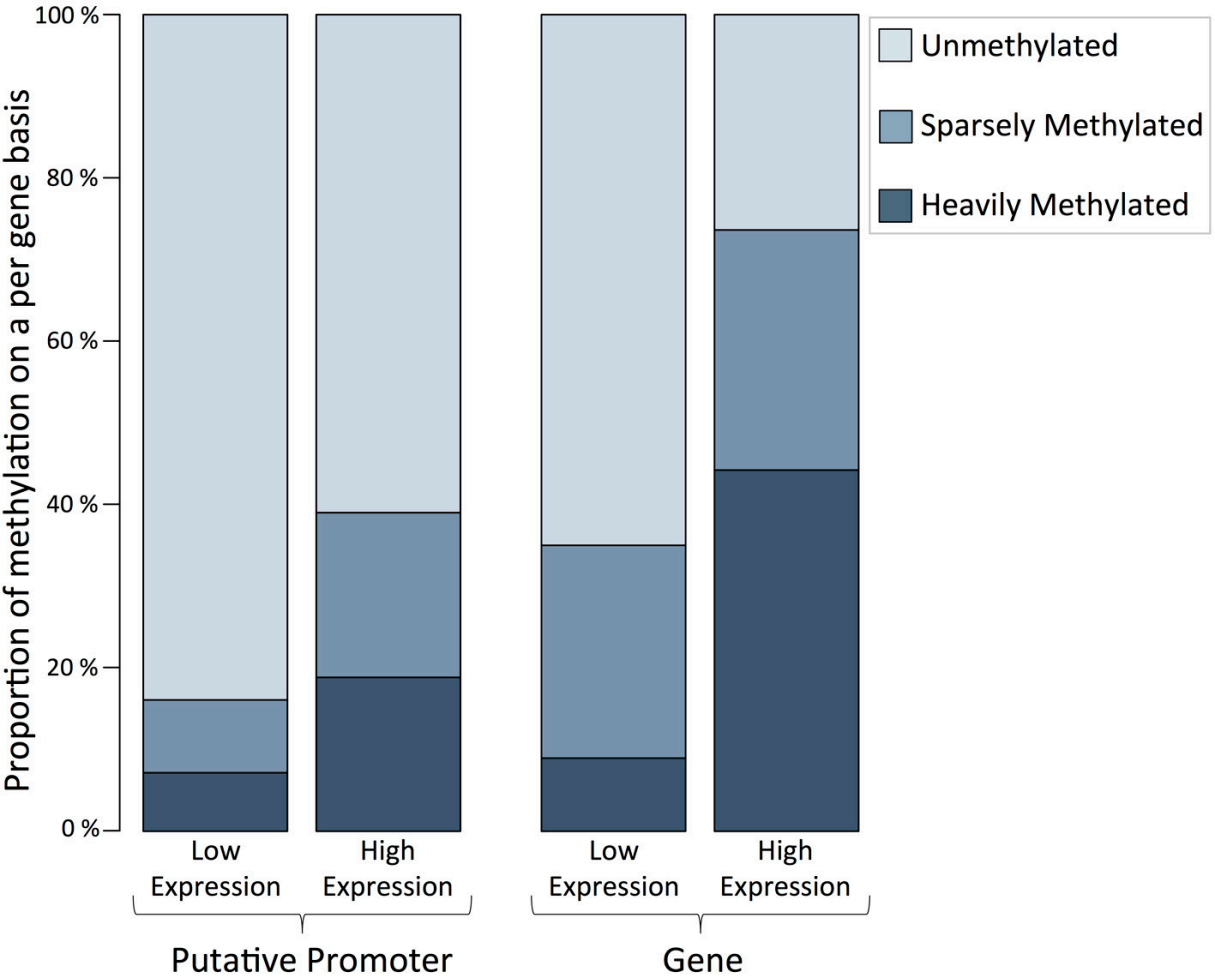
**Proportion of methylation on a per gene basis for putative promoter regions and gene bodies (exon and introns) for high and low expression levels indicate that DNA methylation is positively correlated to gene expression in *C. gigas* male gamete tissue**. Two classifications shown include genes with of low expression (Promoter regions = 13,919 and genes = 14,377, RPKM ≤1, including no expression) and high expression (Promoter regions = 12,477 and genes = 12,877; RPKM > 1). The proportion of corresponding genes that are unmethylated (methylation ratio = 0), sparsely methylated (methylation ratio 0—0.5), and heavily methylated (methylation ratio ≥ 0.5) are shown.

A minimum of one methylated CpG dinucleotide with ≥ 5× coverage was observed for every 14,517 genes in the *C. gigas* genome, or 53% of genes. The proportion of methylated CpGs was characterized with respect to RNA-Seq data on expression levels for full-length genes. Specifically, within genes and putative promoter regions we found a greater proportion of fully methylated CpGs for genes that have elevated expression levels (>1 RPKM) (Figure 3). The observed distributions of methylated CpGs within genes (*X*^2^ = 5493.85, *df* = 2, *p* < 0.0001) and promoter regions (*X*^2^ = 1765.56, *df* = 2, *p* < 0.0001) were significantly different than what would be expected if methylated CpGs were randomly distributed among genes and promoter regions.

Additionally, our transcriptome data was compared to a comprehensive microarray experiment focused on gametogenesis in *C. gigas* (Dheilly et al., 2012). Elevated gene expression was observed in genes identified by Dheilly et al. (2012) to increase in expression over spermatogenesis (clusters 5 and 6) (Olson and Roberts, 2014). Based on this analysis, we suspect our sample was likely from a fully mature male gonad.

## Discussion

Genome-wide shotgun bisulfite sequencing was conducted on gametes from a male oyster to illustrate the role of DNA methylation in a single cell type. While reduced representation CpG DNA methylation has been previously quantified for this species, this study represents the first examination of other dinucleotide motifs. Although CpG DNA methylation is ubiquitous across organisms, types and levels of methylation vary considerably among invertebrates. For example, *Caenorhabditis elegans* essentially lacks DNA methylation in its genome where neither 5-methylcytosine nor DNA methyltransferase are present (Bird, 2002). The *Drosophila melanogaster* genome contains extremely low 5-methylcytosine levels (Gowher et al., 2000; Lyko et al., 2000) and mostly in the CpT dinucleotide context rather than CpG (Lyko et al., 2000). In this study, DNA methylation was only observed in CpG motifs.

This is the first characterization of DNA methylation in bivalve mitochondrial DNA. A lack of methylation in oyster mitochondrial DNA in our study is consistent with previous invertebrate research, which found that genes encoding mitochondrial DNA were unmethylated in the brain tissue of the honey bee *Apis mellifera* (Lyko et al., 2010). Recent research has also demonstrated the absence of CpG methylation in the mitochondrial genome of the sea squirt *Ciona intestinalis* (Suzuki et al., 2013). Studies on the mammalian system have found similar results, confirming a lack of methylation among CpGs in human mitochondrial DNA (Hong et al., 2013).

Overall, approximately 15% of the CpGs are methylated in the oyster male gamete nuclear genome. This is the same estimated proportion from analysis of DNA methylation in oyster gill tissue (Gavery and Roberts, 2013). Similarly, (Suzuki et al., 2013) found identical gene groups to be methylated in tissues from *C. intestinalis* sperm and muscle cells. In their study 23% of the genome was determined to be methylated (Suzuki et al., 2013). The degree of methylation we found in oyster male gamete tissue falls within those previously described for other molluscs. For instance, recent research examining DNA methylation in multiple tissues of the mollusc *Chlamys farreri* found methylation levels in the genome to be around 21% using a methylation-sensitive amplification polymorphism approach (Sun et al., 2014). Studies of DNA methylation in the foot tissue of the gastropod mollusc *Biomphalaria glabrata* found approximately 2% of the CpG dinucleotides to be methylated (Fneich et al., 2013). Our findings, in agreement with previous studies in *C. gigas*, corroborates that overall genome methylation in *C. gigas* is at an intermediate level and suggests that DNA methylation does not significantly vary among tissue type.

DNA methylation is predominantly found in exons and introns in oyster male gametes (Figure 2). These findings are consistent with previous work characterizing DNA methylation in *Crassostrea gigas* gill tissue (Gavery and Roberts, 2013). In several animal and plant genomes, transcribed regions of genes, including intragenic regions, have higher levels of DNA methylation than neighboring regions (Suzuki et al., 2007; Zilberman et al., 2007). DNA methylation in insects, however, appears to be primarily confined to exons (Lyko et al., 2000). Recent work examining DNA methylation in *C. intestinalis* sperm found that methylated domains of the genome are primarily contained within transcription units and promoter regions, with intergenic regions completely unmethylated (Suzuki et al., 2013). Together these studies demonstrate that the distribution of DNA methylation in invertebrate genomes is diverse and that most invertebrate genomes exhibit interspersed regions of methylated and unmethylated DNA. Although we have a relatively comprehensive profile of where DNA methylation occurs across the oyster genome, there is no definitive evidence of a link between DNA methylation and function. However, based on RNA-Seq data there is likely an association between methylation status and gene expression in *C. gigas*.

Recently, Riviere et al. (2013) proposed that proximal promoter and first exon methylation in *C. gigas* larvae have similar functions than those in mammalian systems, where increased methylation in promoter regions corresponds to decreased gene expression. Specifically, Riviere et al. (2013) reported a negative correlation between DNA methylation and expression of some *homeobox* gene orthologs during early oyster development. Our results indicate the opposite- a positive correlation between DNA methylation and gene expression (Figure 3), in which genes with high expression levels had high methylation levels. This is similar to what has been reported for oyster gill tissue (Gavery and Roberts, 2013). Riviere et al. (2013) suggested that a lack of DNA methylation influences gene expression specifically by controlling the transcription level of *homeobox* orthologs in the proximal promoter and first exon. Similarly, the presence of promoter methylation has specifically been associated with transcriptional silencing among many organisms (Suzuki and Bird, 2008). Here we found a significant difference in promoter methylation in high versus low gene expression. It is likely that the relationship of DNA methylation and gene expression is complex and dependent on several factors (Gavery and Roberts, 2014). For example, it has been shown that methylation pattern is dependent on gene function (Roberts and Gavery, 2012) and the current study focused on the entire transcriptome where Riviere et al. (2013) specifically looked at *homeobox* orthologs. The differences we observed between DNA methylation and expression than was previously reported (Riviere et al., 2013) could also be attributable to differences in cell type or a differing role of methylation throughout *C. gigas* life history stages. It should also be noted both studies have limitations as they rely on accurate genome annotation, and, as recently reported (Elsik et al., 2014), this can significantly alter research findings.

Here we provide the first genome-wide characterization of DNA methylation in a bivalve mollusc that focused on a single-cell type. We found that DNA methylation levels in male gametes are similar to those previously reported in gill tissue (Gavery and Roberts, 2013). In examining the relationship between DNA methylation and transcript expression, we observed a pattern suggesting that promoter regions with a higher proportion of CpG methylation are associated with highly expressed genes. Further explorations into the roles of miRNA and histone modifications will help to elucidate our understanding of epigenetic regulatory functions in oysters.

### Conflict of interest statement

The authors declare that the research was conducted in the absence of any commercial or financial relationships that could be construed as a potential conflict of interest.
